# Conventional surgery results in patients originally referred for transcatheter aortic valve implantation

**DOI:** 10.1186/1749-8090-8-S1-O47

**Published:** 2013-09-11

**Authors:** G Rescigno, T Piva, I Mazzanti, C Aratari, G Pupita, A D’Alfonso, A Capucci, GP Perna, L Torracca

**Affiliations:** 1 Departments of Cardiac Surgery, Ospedali Riuniti di Ancona, Ancona, Italy; 2 Academic Cardiology, Ospedali Riuniti di Ancona, Ancona, Italy; 3 Interventional Cardiology, Ospedali Riuniti di Ancona, Ancona, Italy; 4 Cardiology, Ospedali Riuniti di Ancona, Ancona, Italy

## Background

Transcatheter aortic valve implantation (TAVI) is increasingly considered as a viable alternative to conventional aortic valve replacement (AVR) in high-risk patients. However, long term results are still scarce and medical community hesitates in enlarging indications to lower risk subjects. Moreover, available devices are expensive and a strict potential candidate selection is necessary.

## Methods

From April 2008 to August 2012, 212 patients, originally referred for percutaneous treatment, were thoroughly evaluated by the Aortic Team of our Department in order to choose the optimal procedure. Of them 55 patients (35 F;20 M) were considered as still acceptable candidates for conventional AVR.

## Results

Mean age was 80.7 ± 4.7 years, mean additive and logistic Euroscore 1 were 9.7 ± 1.8 and 17.8% ± 9.5, respectively. Mean Euroscore II was 7.9% ± 5.5. Mean NYHA class was 2.9 ± 0.5. The majority of patients (87.2%) presented a geriatric frailty score of 0-1. Four patients showed a heavily calcified ascending aorta and 5 patients (9%) were reoperations. Hospital mortality was 10.9% (6 pts). Mean follow-up was 535.9 ± 407.4 days (range: 6-1365 days). Six other patients died during this period for a mean survival of 74.4% ± 6.9 at 2 years. Mean NYHA class at 1 year was 1.25 ± 0.5 (p< 0.01 vs preoperative value).

## Conclusion

AVR should be indicated with caution in high-risk patients originally referred for TAVI. Despite medium term results are good, with excellent functional status, hospital mortality is not negligible.

